# Discovery of Power-Law Growth in the Self-Renewal of Heterogeneous Glioma Stem Cell Populations

**DOI:** 10.1371/journal.pone.0135760

**Published:** 2015-08-18

**Authors:** Michiya Sugimori, Yumiko Hayakawa, Bruce M. Boman, Jeremy Z. Fields, Miharu Awaji, Hiroko Kozano, Ryoi Tamura, Seiji Yamamoto, Toru Ogata, Mitsuhiko Yamada, Shunro Endo, Masanori Kurimoto, Satoshi Kuroda

**Affiliations:** 1 Department of Integrative Neuroscience, University of Toyama, 2630 Sugitani, Toyama, Toyama 930–0194, Japan; 2 Department of Neurosurgery, University of Toyama, 2630 Sugitani, Toyama, Toyama 930–0194, Japan; 3 Center for Translational Cancer Research, Departments of Biology and Mathematics, University of Delaware, Helen F Graham Cancer Center and Research Institute, Newark, DE 19711 United States of America; 4 Biotechnical Research, CATX, Inc., Gladwyne, PA 19035 United States of America; 5 Department of Pathology, University of Toyama, 2630 Sugitani, Toyama, Toyama 930–0194, Japan; 6 The Center of Sports Science and Health Promotion in the NRCD Hospital, National Rehabilitation Center for Persons with Disabilities, 4–1 Namiki, Tokorozawa, Saitama 359–8555, Japan; 7 Department of Neuropsycopharmacology, National Institute of Mental Health, National Center of Neurology and Psychiatry, 4-1-1 Ogawahigashi, Kodaira, Tokyo 187–8553, Japan; National Cancer Institute, UNITED STATES

## Abstract

**Background:**

Accumulating evidence indicates that cancer stem cells (CSCs) drive tumorigenesis. This suggests that CSCs should make ideal therapeutic targets. However, because CSC populations in tumors appear heterogeneous, it remains unclear how CSCs might be effectively targeted. To investigate the mechanisms by which CSC populations maintain heterogeneity during self-renewal, we established a glioma sphere (GS) forming model, to generate a population in which glioma stem cells (GSCs) become enriched. We hypothesized, based on the *clonal evolution* concept, that with each passage in culture, heterogeneous clonal sublines of GSs are generated that progressively show increased proliferative ability.

**Methodology/Principal Findings:**

To test this hypothesis, we determined whether, with each passage, glioma neurosphere culture generated from four different glioma cell lines become progressively proliferative (i.e., enriched in large spheres). Rather than monitoring self-renewal, we measured heterogeneity based on neurosphere clone sizes (#cells/clone). Log-log plots of distributions of clone sizes yielded a good fit (r>0.90) to a straight line (log(% total clones) = k*log(#cells/clone)) indicating that the system follows a power-law (y = x^k^) with a specific degree exponent (k = −1.42). Repeated passaging of the total GS population showed that the same power-law was maintained over six passages (CV = −1.01 to −1.17). Surprisingly, passage of either isolated small or large subclones generated fully heterogeneous populations that retained the original power-law-dependent heterogeneity. The anti-GSC agent Temozolomide, which is well known as a standard therapy for glioblastoma multiforme (GBM), suppressed the self-renewal of clones, but it never disrupted the power-law behavior of a GS population.

**Conclusions/Significance:**

Although the data above did not support the stated hypothesis, they did strongly suggest a novel mechanism that underlies CSC heterogeneity. They indicate that power-law growth governs the self-renewal of heterogeneous glioma stem cell populations. That the data always fit a power-law suggests that: (i) clone sizes follow continuous, non-random, and scale-free hierarchy; (ii) precise biologic rules that reflect self-organizing emergent behaviors govern the generation of neurospheres. That the power-law behavior and the original GS heterogeneity are maintained over multiple passages indicates that these rules are invariant. These self-organizing mechanisms very likely underlie tumor heterogeneity during tumor growth. Discovery of this power-law behavior provides a mechanism that could be targeted in the development of new, more effective, anti-cancer agents.

## Introduction

Despite decades of intense research, few advanced cancers are cured by chemotherapy. One possible explanation is that a malignant tumor is composed of multiple cell types and that in chemotherapy the wrong subtypes of cells are being targeted. Current opinion has been increasingly suggesting that cancer stem cells (CSCs) may be the right subtype [1,2]. In that view, growth and progression of cancers are now thought to be driven by CSCs [1–6], whereby a small sub-population of homogeneous, tumor-propagating cells continuously generates all the other cells of a malignant tumor. This is why most chemotherapeutic agents are designed to target rapidly dividing cancer cells that constitute the bulk of the tumor. While many types of CSCs are known to be quiescent, brain cancer researchers have shown that glioma SCs are proliferative. This provides a likely mechanism as to why more advanced cancers are not cured [1,2,7–10].

In targeting CSCs, however, there may be complications due to the cellular makeup of tumors. Although many early studies using specific molecular markers identified CSCs as a homogeneous population, more recent studies suggest that the CSC population is heterogeneous rather than homogeneous, as previously suspected [9,11–18]. Given this heterogeneity, it remains unclear how CSCs might be effectively targeted. For one thing, during cancer growth CSCs might reversibly change their phenotypes. Indeed, recent studies [19] show that there is inter-conversion between different cell subtypes within tissues, including cancer tissues. This would lead to varying sensitivities of cells within a cancer, including CSCs, to radiation and systemic agents [11]. For another, phenotype inter-conversion might provide the targeted CSCs a way to evade agents designed to target a particular CSC subset.

Yet another conceptual issue arises: if a malignant tumor contains heterogeneous CSCs with differing frequencies of self-renewal, then those CSC clones that self-renew most rapidly would, through competition with other CSCs, be predicted to eventually account for the majority of the total CSC population. This could lead to a loss of CSC heterogeneity which raises key questions that are addressed in the current study–Is CSC heterogeneity actually maintained in a cancer cell population? If it is maintained, how do heterogeneous CSC sub-populations self-renew?

Consequently, we studied the mechanism by which CSC populations might maintain heterogeneity during their self-renewal. In particular, if the mechanism leads to heterogeneous CSC clones that have different proliferative potential, then the clone sizes would become different over time. For example, a study [20] of skin tumors reported that the population of tumor cells consisted of clones with different numbers of cells per clone and that the differentials in cell numbers between clones was established by terminal differentiation of tumor cells programmed with different timings. This finding raises another important question addressed in our study: If a CSC population is heterogeneous, are the differential sizes of clones established by terminal differentiation in clones, or by differential times of cell cycling in conjunction with symmetric or asymmetric self-renewal of CSCs.

The approach to answering these questions that we took was to study malignant cells derived from those tumors because they were known to contain CSCs [21–23]. We chose to study gliomas [24] because the glioma model represents a robust system for studying tumor SC heterogeneity. Indeed, recent studies have reported that glioma stem cells (GSCs) are heterogeneous [25,26]. But, how a heterogeneous GSC population self-renews has not been reported. To begin to fill this gap in our knowledge, the present study was conducted using tumor neurosphere cultures in which GSCs are clonally grown and enriched to form tumor-cell-derived neurospheres (glioma spheres; GSs). The ability to form such neurospheres is often used as a measure of stemness. Here, we used in vitro clonal analysis of GS populations to assay how CSC populations maintain heterogeneity during their self-renewal. In the present study, we refer to these GSs as glioma stem cell (SC)-like cells because they exhibit self-renewal properties over multiple passages in neurosphere cultures.

Based on the *clonal evolution model* it would be predicted that, during tumor progression, genetic variants are continuously produced in which the growing neoplasm contains mutant clonal sublines that have a continuously increasing ability to proliferate and a continuously decreasing ability to differentiate [27]. Thus, we hypothesized that, with each passage in culture, heterogeneous clonal sublines of GSs are generated that progressively show an increased ability to proliferate as demonstrated by progressive enrichment of large spheres with each passage in culture.

## Materials and Methods

### Tumor neurosphere culture

#### The Clonal Assay

The cell lines A172, T98G, U251, and U87 were selected for study because of their sphere-forming ability and because these lines have been molecularly classified based on the common glioma biomarkers [28]. The lines were obtained from the following sources: A172 (JCRB0228, Japanese Collection of Research Bioresources: JCRB, Osaka, Japan; CRL1620, American Type Culture Collection: ATCC, Rockville, MD, USA), T98G (CRL1690, ATCC), U87 (RCB419, RIKEN BioResource Center, Tsukuba, Japan; HTB-14, ATCC) and U251 (RCB0461, RIKEN BioResource Center). The growth medium consisted of Dulbecco’s modified eagle medium/F-12 (Life Technologies, Carlsbad, CA, USA) with 1/50 of retinoic acid-free B27 (Life Technologies), 1 mg/ml bovine serum albumin (Sigma-Aldrich, St. Louis, MO, USA), 20 ng/ml basic fibroblast growth factor (Life Technologies) and 20 ng/ml epidermal growth factor (Life Technologies) [29–31]. To develop tumor-cell-derived neurospheres, single glioma cell line-derived cells were cultured under non-adherent conditions using poly(2-hydroxyethyl methacrylate) (polyHEMA, Sigma-Aldrich) coated 100 mm dishes (BD Biosciences, Franklin Lakes, NJ, USA) (100,000 cells/ml). Then, the cell line-derived glioma spheres (GSs) were measured as surviving clones. To determine the frequency (i.e., the proportion) of self-renewing GSs in the population, cells were dissociated with 0.1% trypsin + 400 μM EDTA (Life Technologies) and seeded in growth medium containing 0.8% methylcellulose (Nacalai Tesque, Kyoto, Japan) on day 0 in poly-HEMA coated 6-well plates (BD Biosciences) at a clonal density of (10,000 cells/5 ml/well). Numbers of clones and numbers of cells per clone were quantified under a 20x objective lens light microscope (Nikon, Tokyo, Japan), and assessed on days 1, 4 and 7.

#### Repopulation experiments

U87-derived GS clones were developed in 100 mm dishes as described above. Tumor neurosphere-containing populations were passaged six times. Cells derived from each passage were subject to clonal assays. Each series of repopulation experiments were repeated three to five times.

#### Fractionation experiments

Clones, which were developed for 14 days in the methylcellulose-containing growth medium to separate readily expanded (big) clones from less expanded (small) clones, were recovered and filtered through a 40 μm cell-strainer (BD Biosciences). After both the retained fraction (L fraction, which contains well-expanded large-sized clones) and the passed fraction (S fraction, which contains less-expanded small-sized clones) were dissociated with trypsin into single cells, the cells were clonally seeded and assayed in the methylcellulose matrix at days 1, 4 and 7. The clones in both the L and S fractions were recovered at day 14, and then filtered to separate retained clones (LL fraction from the L fraction, SL from S) and passed clones (LS from L, SS from S). The clones were again dissociated into single cells to conduct the clonal assay.

#### Administration of Temozolomide in tumor neurosphere culture

We planned two different experiments for Temozolomide administration: i) 25, 125, 625 μM of Temozolominde was administered in the assaying culture, then the number of cells in each clone was quantified in the presence (25, 125, 625 μM) and in the absence (0μM) of Temozolomide. ii) U87-derived GS clones were cultured and allowed to develop tumor neurospheres in the presence of Temozolomide, and then the clones were dissociated in a subsequent generations. The dissociated clones were seeded and allowed to develop in the absence of Temozolomide, and then we analyzed each clone size. The above two experiments were repeated six (1st to 6th) generations for the former experiment and three (2nd to 4th) generations for the latter.

### Graphs and statistical analyses

All graphing and regression analyses were done using GraphPad Prism version 5.0b for Mac OS X (GraphPad Software, Inc, San Diego California USA, www.graphpad.com.). To analyze the diversity in clone sizes generated by the GS population, we developed a quantitative index. To this end, we calculated the coefficient of variation (CV) in the number of cells per clone. CV is the ratio of the standard deviation s to the mean m: CV = s/m.

## Results

### I. Growth Properties of GSs

#### Diversity in the growth of GSs

**Fig 1:**
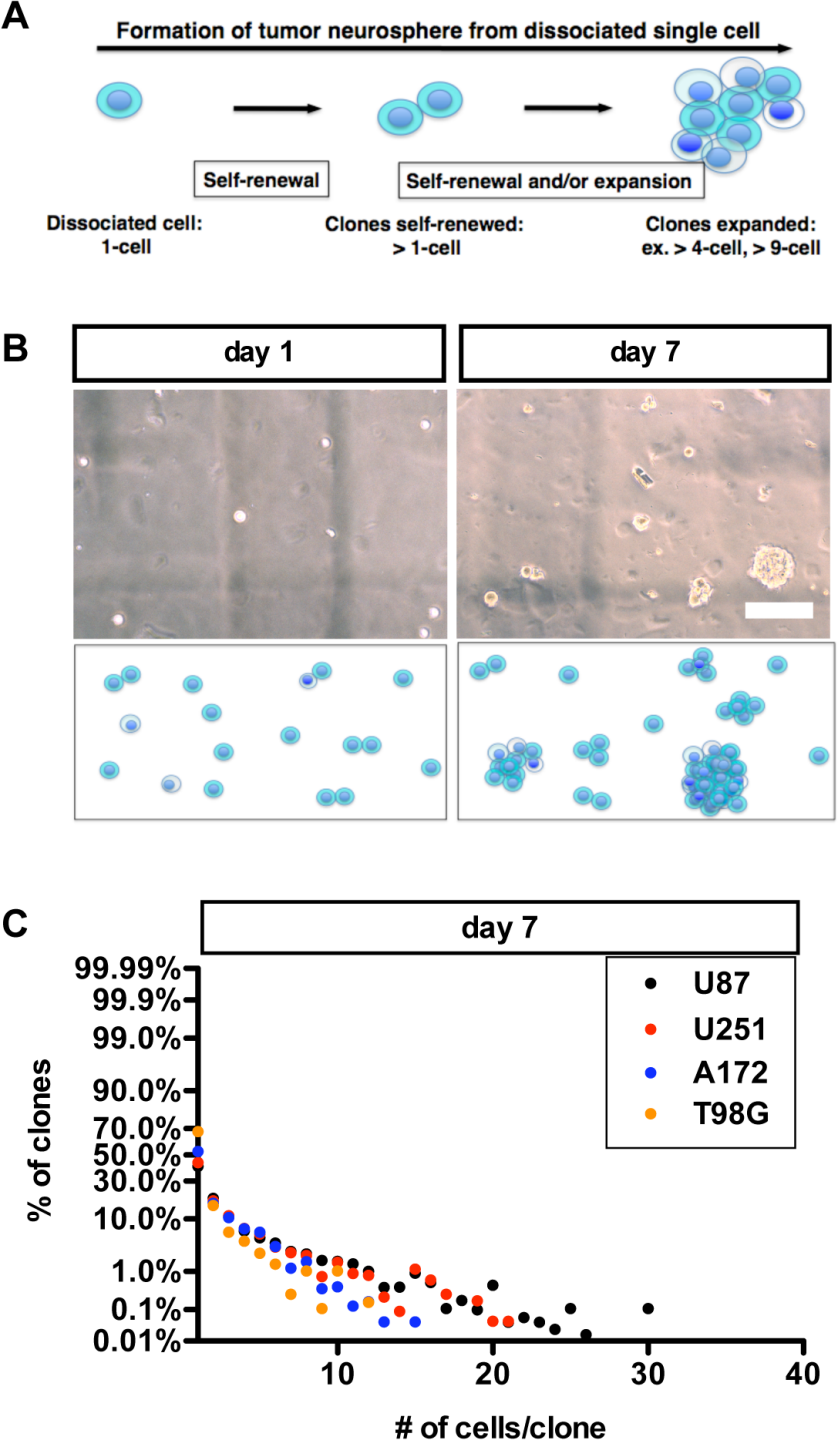
Growth diversity of glioma cell line-derived GSs in the tumor neurosphere culture. We seeded glioma cell line-derived GSs in methylcellulose-containing growth medium at an initial clonal density of 10,000 cells/ 5 ml. (A) A schematic diagram of tumor neurosphere formation from a single cell clone. (B) U87 derived GS clones in the tumor neurosphere culture. A representative picture and schematic diagram of the population at day 1 (left) and 7 (right). Scale bar = 200μm. (C) Diversity for growth in the population of U87 (circles and the regression line in black); U251 (in red); A172 (in blue); T98G (in orange) derived GS clones. The frequency of clones of different size (number of cells per clone) at day 7 in a GS population (C) is shown.

We quantitatively determined the diversity in clone sizes (1–40 cells/clone) that were generated by GSs. The GSs were undifferentiated cells that had been derived from four different glioma-derived cell lines including U87, U251, T98G and A172, which were seeded in colony-forming assays. On day 7, we observed (Fig 1A) that every GS clone that had been derived from an undifferentiated cell line generated clones that had a wide variety of clone sizes. We then assessed the number of clones and the number of cells per clone at days 1, 4 and 7 (Fig 1B). The number of cells per individual clone reflects growth properties such as rate of growth or potential for self-renewal and clonal expansion. Using our quantitative index (CV) to assess diversity in clone sizes generated by the GS population, we found that the CV that was the highest among the four cell-lines tested was 1.03 at day 7 for the U87-derived GSs (Fig 1C). A higher CV suggests a greater diversity in the numbers of cells per clone. We then characterized the self-renewal and clonal expansion properties of each of the four cell lines.

#### Survival and differential self-renewal activity of clones

Approximately 40% of the clone population survived throughout the culture period (days 1 to 7) (S1A Fig), indicating that a substantial proportion of GSs remain in the culture system. If single-cell (1-cell) clones never self-renewed, the number of 1-cell clones would remain constant. On the other hand, when 1-cell clones undergo cell division in which the clones symmetrically generate stem cells (1 SC → 2 SC), or asymmetrically generate a non-SC while maintaining the size of the SC population (1 SC → 1 SC + 1 non-SC), the number of cells in each clone increases. The variables that reflect self-renewal of clones are both the decrease in the number of 1-cell clones and the increase in the number of clones with multiple cells (>1-cell clones). From day 1 to day 4, we observed an increase in the number of >1-cell clones with a simultaneous decrease in the number of 1-cell clones (S1A Fig). This indicates that self-renewal within the GS clone population increased monotonically. We then calculated the percentage of self-renewed clones among the total remaining population. By day 1, 30% of the surviving clones had self-renewed. This proportion increased to ~55% by days 4 and 7. In parallel, the percentage of 1-cell clones decreased from ~70% to ~45% (S1B–S1C and S1F–S1G Fig). Thus, we can conclude that the number of, and the percentage of, self-renewed clones were not significantly affected by cell death.

#### Differential expansion of clones

Both the number and percentage of >4-cell and >9-cell clones among the total clone population monotonically increased (S1D–S1E and S1H–S1I Fig). The percentage of expanded clones (e.g., >4-cell or >9-cell) among the self-renewed clones (>1-cell clones) is a more precise reflection of clonal expansion capacity (S1J and S1K Fig). The data again showed increases in the percentages of >4-cell clones vs. >1-cell clones, >9-cell clones vs. >1-cell clones and >9-cell clones vs. >4-cell clones (S1J–S1L Fig). Thus, with repeated self-renewal, the GS clones consistently expanded to form tumor neurospheres.

#### Total cell number represents the population size of diverse GSs

The total number of GSs in a population–determined by adding together the number of cells in each individual clone, or by multiplying the average number of cells in each clone by the total clone number–is a key indicator of tumor cell expansion. That number significantly increased from day 1 to day 4, and stayed relatively constant from days 4 to 7 (S1M–S1P Fig; S2A–S2E Fig). Clonal expansion (an increase in # cells / clone) monotonically increased from days 1 to 7 (S1D and S1E; S1H and S1I; S1J–S1L Fig). As noted above, the GS clones grew at diverse rates, but the average number of cells in each clone increased consistently from days 1 to 7 (S1M–S1P Fig). Thus, the increase in the number of GSs in the neurosphere culture indicated that the overall GS population grew over the seven days.

#### The number of cells in single, self-renewed and expanded clones within the total GS cell population

We next calculated how much of the total GS population was derived from single, self-renewed or expanded clones. The number of cells originating from 1-cell clones decreased, while the number of cells originating from >1-cell clones increased (S2A–S2C Fig) as self-renewal occurred continuously. The number of cells in >4-cell and >9-cell clones also increased during the culture period (S2E and S2E Fig).

We then determined how much self-renewal and clonal expansion occurred as part of the growth of the GS population. About 50% of clones stayed as 1-cell clones, and that percentage remained constant from days 4 to 7 (S1B–S1C and S1F–S1G Fig), suggesting that 1-cell clones no longer actively self-renewed. The percentage of cells from 1-cell clones decreased from 51% to 16% (Fig F in S2 Fig), while the percentage of cells from >1-cell clones increased from 49% to 84% (S2G Fig). This suggests that from days 4 to 7 the increase in the total cell population is attributable to growth among self-renewed clones. This idea is supported by the significant increases in the percentages of >4-cell and >9-cell clones (S2D–S2E and S2H–S2I Fig). We also found that the percentages of cells from >4-cell and >9-cell clones increased gradually (S2H and S2I Fig). Furthermore, fractions were shifted from the population of clones with 2–4 cells to clones with 5–9 or >9 cells within the self-renewed clone-derived population (S2J–S2L Fig). Thus, within the GS population, self-renewed clones grew the most.

### II. The Diverse Growth of Clones Follows a Power-Law

**Fig 2:**
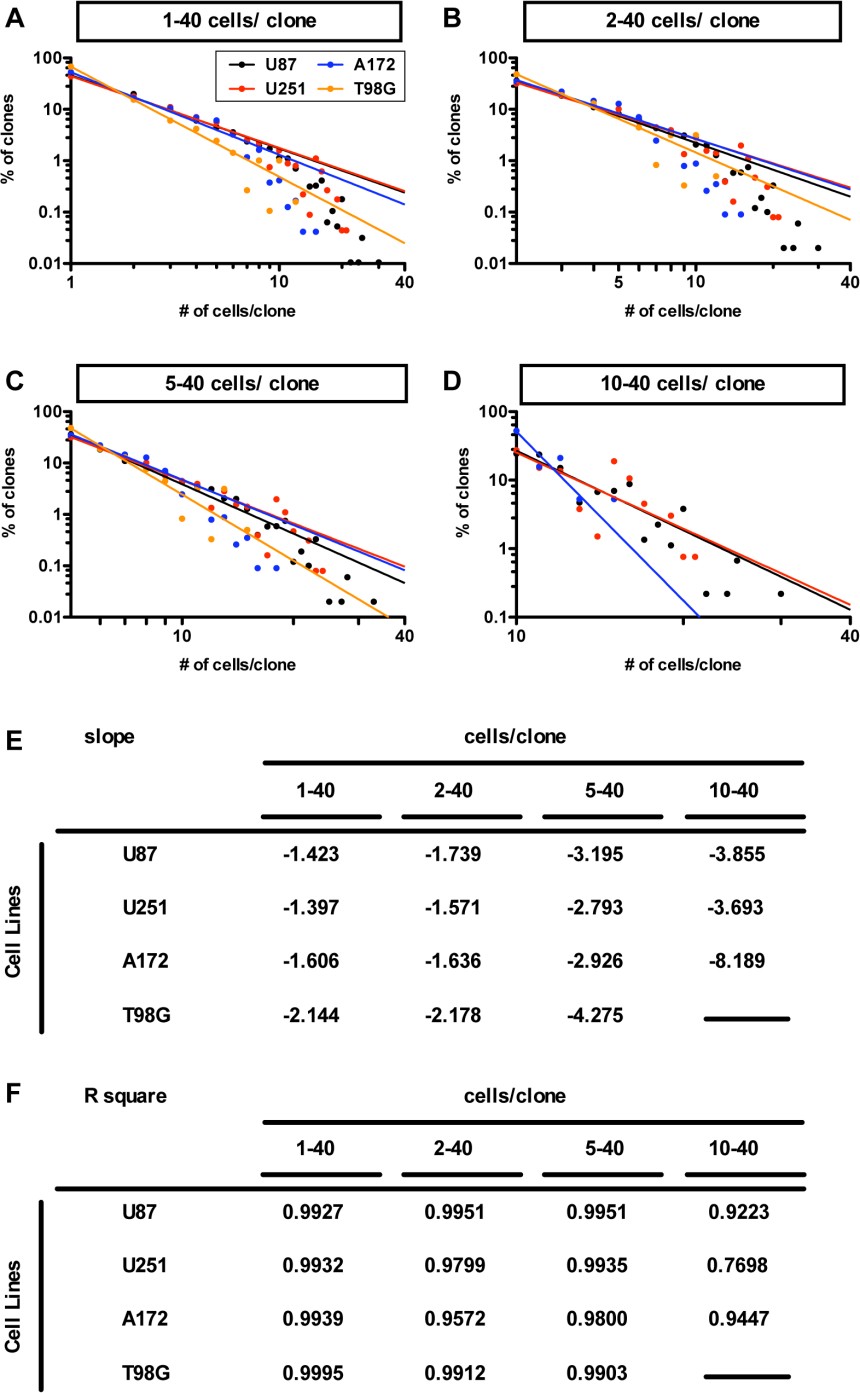
Power-law in frequency distribution of glioma cell line-derived GSs in the tumor neurosphere culture. (A)–(D) Power-law for growth in the population of U87 (circles and the regression line in black); U251 (in red); A172 (in blue); T98G (in orange) derived GS clones. The graphs show the double logarithmic plot of the size and the frequency of the GS clones at day 7 for the population of all clones (1–40 cells/clone for A), of self-renewed clones (2–40 cells/clone for B), of clones with five to forty cells (5–40 cells/clone for C) and of clones with ten to forty cells (10–40 cells/clone for D). (E)-(F) The tables show slopes (E) and R values squared (F) of the regression lines of (A)-(D).

Using our quantitative index (CV) to assess diversity in clone sizes generated by the GS population (Fig 1C), we also found that the x and y variables appeared to be exponentially related (and not follow a bell-shaped curve, i.e., not normally distributed). This suggested the possibility that a power-law might be involved. To test this possibility, we plotted the frequency distribution of the number of cells per clone vs. % of clones on a log-log plot (Fig 2A–2D). Indeed, the data appeared to best fit a straight line, where the coefficient of determination (R^2^), the coefficient of correlation squared, was more than 0.99 in all plots of cell line-derived GSs. This suggests that the clonal diversity that accompanies clonal expansion follows a power-law of the form log (y) = k * log (x), and y = x^k^, where k, the degree exponent, is a constant. Here, the numerical values of k (k_1–40_) were found to be −1.423, −1.397, −1.606, −2.144 for U87-, U251-, A172- and T98G-derived GSs, respectively. Thus, across all cell lines tested, the distribution of clone sizes follows a power-law. This suggests that the heterogeneity in the size of clones is not randomly formed; rather the heterogeneous growth of a GS population appears to be governed by a mechanism that follows a power-law. Indeed, the most frequent clone size was the single cell (i.e., cells that were not self-renewed). This is consistent with the idea that the high frequency of single cell-clones is also encoded by a power-law.

We then determined whether self-renewed clones followed a power-law. Hence, we graphed, on a log-log plot, data for the population of clones derived from self-renewed clones (those containing two or more cells in each clone). The frequency distribution of self-renewed clones also appeared to follow a power-law (R^2^: 0.995, 0.980, 0.957, 0.991; k_2–40_: −1.739, −1.571, −1.636, −2.178 for U87-, U251, A172, T98G-GSs, respectively in Fig 2B, 2E and 2F). The frequency distribution generated by large clones, ones that had >4 cells in each clone (5–40 cells in Fig 2C, 2E and 2F; R^2^: 0.995, 0.994, 0.980, 0.990; k_5–40_: −3.195, −2.793, −2.926, −4.725 for U87-, U251, A172, T98G-GSs, respectively) or >9 cells in each clone (10–40 cells in Fig 2D, 2E and 2F; R^2^ = 0.922, 0.768, 0.945; k_10–40_ = −3.855, −3.693, −8.189 for U87-, U251, A172-GSs, respectively), also followed a power-law.

These findings suggest that the power-law that governs the growth of clones of every glioma cell line-derived GS population generates clones of diverse (heterogeneous) sizes, and is scale-free. We even found evidence of a scale free power-law in self-renewed clones: the absolute number of k was higher in populations of larger clones showing that larger clones are more infrequent.

### III. Reproducibility of diversity and power-law behavior during repopulation of GSs

#### Recovery of diversity in the tumor neurosphere culture during repopulation

We next addressed the following question: During repopulation of the culture, does (i) the GS population size grow while maintaining GS heterogeneity and clonal diversity, or do (ii) GSs follow a clonal evolution model in which expandable clones grow more robustly and replace the previously dominant population of clones?

**Fig 3:**
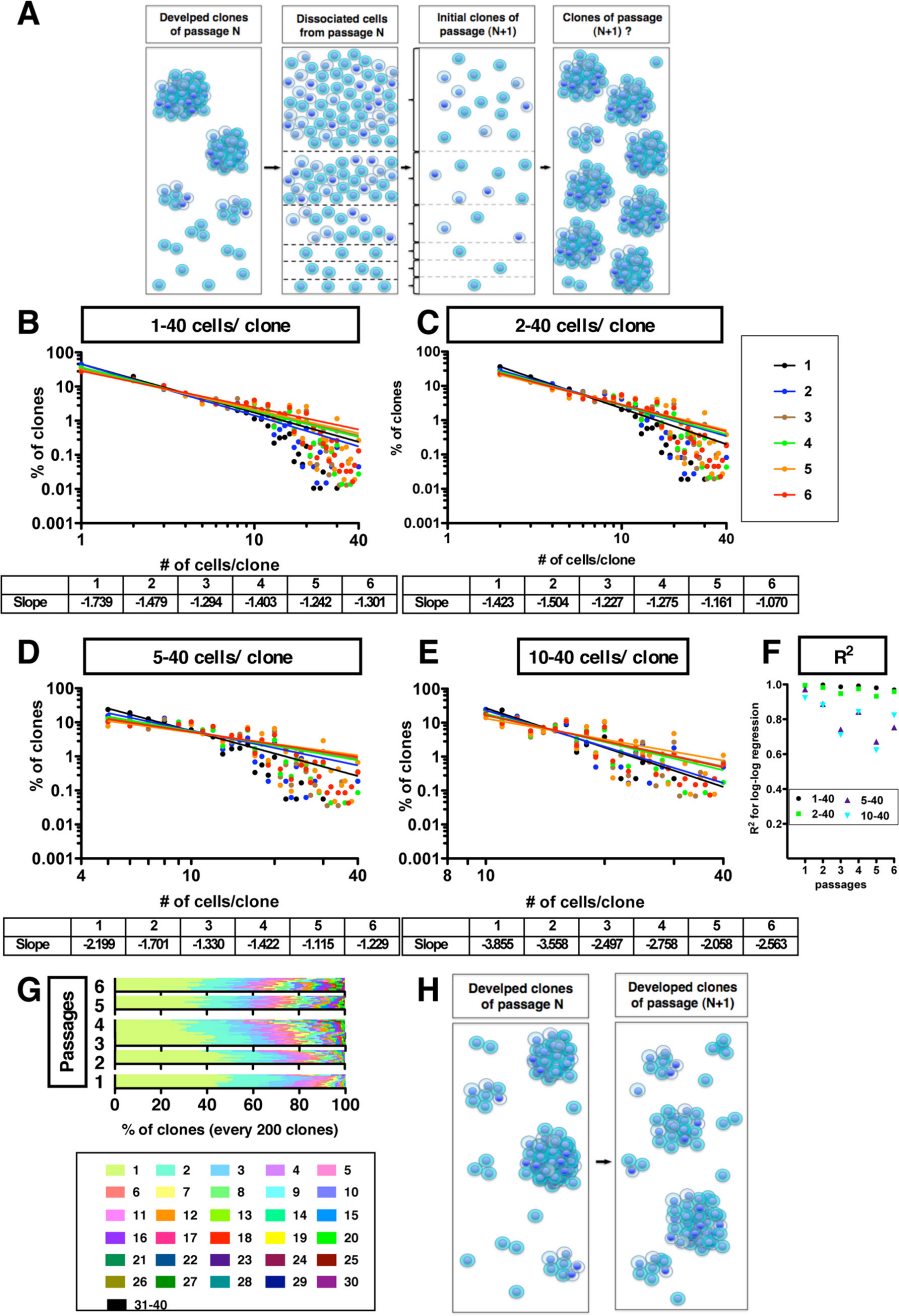
Recapitulation of power-law growth during repeated passages. (A) The diagram shows enrichment of dominant clones, and clonal evolution. (B)-(E) The panels show a double logarithmic plots of the clone size (number of cells in each clone) and the frequency of the GS clones at day 7 for the population of all clones (B), of self-renewed clones (C), of clones with five to forty cells (D) or of clones with ten to forty cells (E). Lines in (B-E) are regression lines. (F) The R^2^ values for the Log-Log Regression lines for (B-E) are shown. (G) Bar graphs in each passage experiment show the compositions of every two hundred diverse clones at day 7. The range of quantified clones in the passage experiments was 5291–9489 clones. Colored patterns, which indicate the number of cells per clone, are listed. (H) The diagram shows the summary of repopulation experiments.

Based on the latter [27], it would be predicted: (i) that with each passage in culture, heterogeneous clonal sublines of GSs are generated that progressively show an increased ability to proliferate, (ii) a loss of diversity in clone size, reflecting (iii) a loss of heterogeneity in the GS population (Fig 3A).

Accordingly, we determined if the diversity in clone sizes (#cells/clone) was maintained during repeated passages. Because, of the glioma cell lines tested, the U87-derived GSs showed the greatest diversity and the greatest frequency of the appearance of clones, we thereafter used only U87 cells.

After every passage, a double logarithmic plot of the frequency distribution of the number of cells per clone (Fig 3B) showed a major population of small clones and a minor population of big clones. This shows that replacement of small clones by large clones did not occur. We consistently found that cell populations did not show significant differences from one passage to the next (S3A Fig). Indeed, the CV values did not show significant differences during repeated passages (CV values of 1.03–1.17 at day 7), showing that the specific pattern of growth diversity (i.e., the heterogeneity in the population) was recapitulated (S3B Fig). These data show that the growth characteristics of GSs are retained during repopulation of the total GS population and do not support the clonal evolution model, at least not with regard to SC heterogeneity.

#### Recovery of the power-law for growth during repopulation

When we repeated passaging of the GS population, we found that the power-law for clonal growth was maintained during repopulation (Fig 3B–3E). The coefficients of determination (R^2^ values) remained around 0.96–0.99 in the population of total clones and in the population of self-renewed clones during all six passages (Fig 3F). This indicates that the power-law-based growth relationship in the population was maintained over 6 passages. The k value for the total population (k_1–40_) was −1.42 for the first passage. From the 2^nd^ passage on, k_1–40_ decreased passage by passage (from—1.50 to −1.07). k_2–40_, k_5–40_ and k_10–40_ also became smaller, indicating that the culture system became enriched in expandable clones while maintaining power-law growth (k_2–40_: −1.74 to −1.30; k_5–40_: −2.20 to −1.30; k_10–40_: −3.86 to −2.56; corresponding R^2^ values are shown in Fig 3F).

#### Space-free recapitulation of diversity and power-law for growth

Because it was unclear how many clones are required to recapitulate a growth power-law, we determined if a part of the GS population, 200 clones (about 10 visual fields), exhibits diversity in clonal growth. Sub-populations of 200 clones showed a range of clone sizes. Indeed, the frequency distribution of clone sizes in the populations generated from every sample of 200 clones appeared similar to the distribution in the entire population (Fig 3G), suggesting that even parts of the population show similar diversity in growth, and, that the power-law is not dependent on the space occupied by the initial population. Moreover, when we repeated passages of GSs, the clonal diversity of the different regions of the total population was recapitulated (Fig 3G), suggesting that the space-free diversity and power-law controlling clonal growth is not restricted over multiple passages. Thus, the diversity in sizes of clones and the underlying power-law for clonal growth are space-free and maintained during repopulation, and it appears that the diverse GS populations self-renewed (Fig 3H).

### IV. Plasticity in the self-renewal of GSs

#### Reconstruction of diversity during repopulation of both small- and large-sized clones

**Fig 4:**
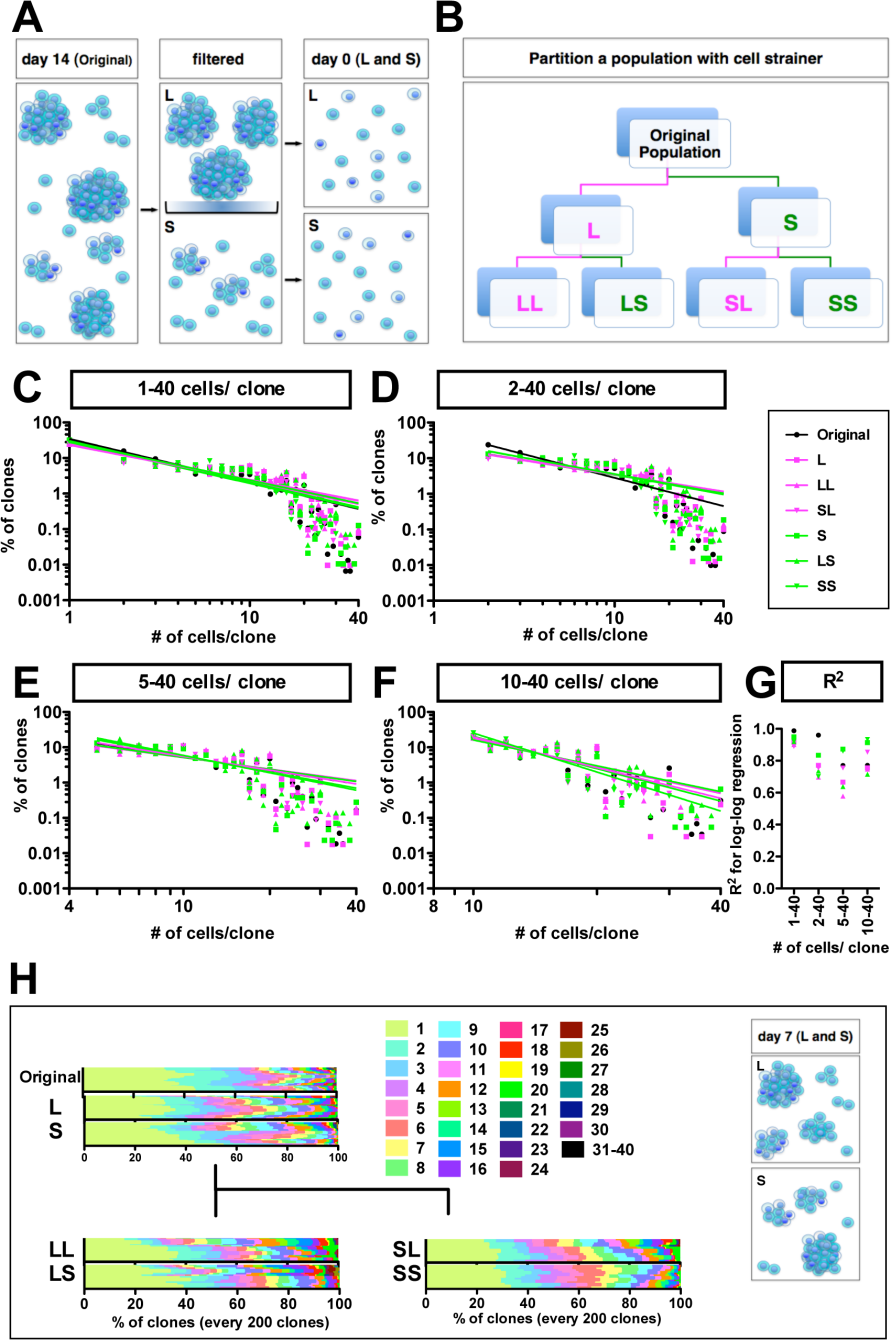
Recapitulation of power-law growth during repopulation of both large- and small-sized clones. (A) Schematic diagram of separation of clones by filtration. The population of clones developed for 14 days were filtered with a 40 μm cell strainer. Clones in both large retained (L fraction) and small passed (S fraction) were dissociated into single cells with trypsin. The cells were then clonally seeded in the methylcellulose matrix. (B) The strategy for how the original population was partitioned. The original population of clones was partitioned into L and S fractions. Clones derived from both L and S fractions were developed for 14 days and partitioned into LL and LS sub-fractions from the L fraction, and into SL and SS sub-fractions from the S fraction. (C) The graph shows a double logarithmic plot of the clone size (number of cells in each clone) and the frequency of the GS clones at day 7 for each separate population (C-F); of all clones (C); of self-renewed clones (D); of clones with five to forty cells (E); of clones with ten to forty cells (F). Sines in (C-F) are regression lines for the double logarithm plots. The R^2^ values for the log-log regression lines in (C) are 0.99, 0.92, 0.90, 0.89, 0.95, 0.92 and 0.92 for the original, L, LL, SL, S, LS and SS fractions shown in (G). (H) Bar graphs in each experiment show the composition of every two hundred diverse clones at day 7. The number of quantified clones in each separate experiment ranged from 2098 to 10392 clones. Colored patterns, which indicate the number of cells per clone, are listed. A summary diagram is shown.

Here, we determined if the expanded clones could, by themselves, reconstruct clonal diversity in a population. GSs were cultured for 14 days, and about 70% of the clones became tumor neurospheres that were over 100 μm in diameter. We then filtered the population with a 40μm cell strainer to separate larger clones (L, LL and LS fractions, which were retained on the filter) from smaller clones (S, SL and SS fractions, which passed through the filter) (Fig 4A and 4B). We found that 60% of the stuck clones contained more than 15 cells (an average of 102 cells/clone) and 90% of clones that passed contained fewer than 15 cells (an average of 5 cells/clone). We then dissociated the tumor neurospheres in the L fraction and conducted clonal assays. The frequency distribution again showed high percentages of smaller-sized clones and low percentages of expanded clones (CV: 0.89 for the L fraction; S4A and S4B Fig), suggesting that GSs establish clonal diversity during clonal expansion.

We next examined whether smaller clones also generate clonal heterogeneity (including both large and small clones). Surprisingly, cell populations (S) derived from smaller clones generated diversity in clone size (CV: 0.97 for the S fraction; S4A and S4B Fig). Moreover, we repeated the passaging experiments and again found that both larger (LL and SL) and smaller (LS and SS) clone-derived cell populations exhibited diversity (CV values of 0.89, 0.84, 0.94 and 0.85, respectively, for LL, SL, LS and SS fractions; S3B Fig). This suggests that individual fractions of the GS population are able to re-establish a diverse population (clonal heterogeneity) repeatedly. It appears that GSs flexibly and reversibly change their growth properties when the GS population self-renews through repeated passaging.

#### Recapitulation of the power-law for growth during repopulation of both small and large clones

Log:Log plots showed that the power-law for growth was re-established during repopulation of both larger (L, LL and SL) and smaller (S, LS and SS) sized clone-derived neurospheres (Fig 4C–4F). R^2^ values remained around 0.89–0.99 in the original-, L-, LL-, SL-, S-, LS-, and SS- derived populations (Fig 4G). The data show that growth of populations derived from each fraction follows a power-law with k values ranging from −0.97 to −1.22. We then determined whether k values were different in large clones (L, LL, SL), small clones (S, LS, SS), original-derived (original, L, LL, LS or original, S, SL, SS), L-derived (L, LL, LS) and S-derived (S, SL, SS) populations. The values of k_1–40_ were not significantly different (large clones: −1.00 + 0.05; small clones: −1.13 + 0.05; original-derived L: −1.10 + 0.11; original-derived S: −1.11 + 0.11; original-derived both L and S: −1.09 + 0.10; L-derived: −1.07 + 0.09; S-derived: −1.07 + 0.10). This suggests that power-law growth is retained in both small and large clone-derived populations, leading to the idea that recapitulation of clonal heterogeneity is not dependent on clone size.

To examine scale-free power-law growth properties, we studied sub-populations containing two hundred cells. These subpopulations generated a pattern of clonal diversity that was similar to the patterns for the original populations from which they were derived (Fig 4H). This similarity suggests that power-law growth is not restricted by the location of the GS population in a local field. Thus, the GS population self-renews via a mechanism that involves recapitulation of a scale-free and space-free power-law growth mechanism.

### V. Effects of the glioma-targeting agent Temozolomide

#### Recapitulation of the power-law for growth during development of U87-derived GS clones in the presence of Temozolomide

**Fig 5:**
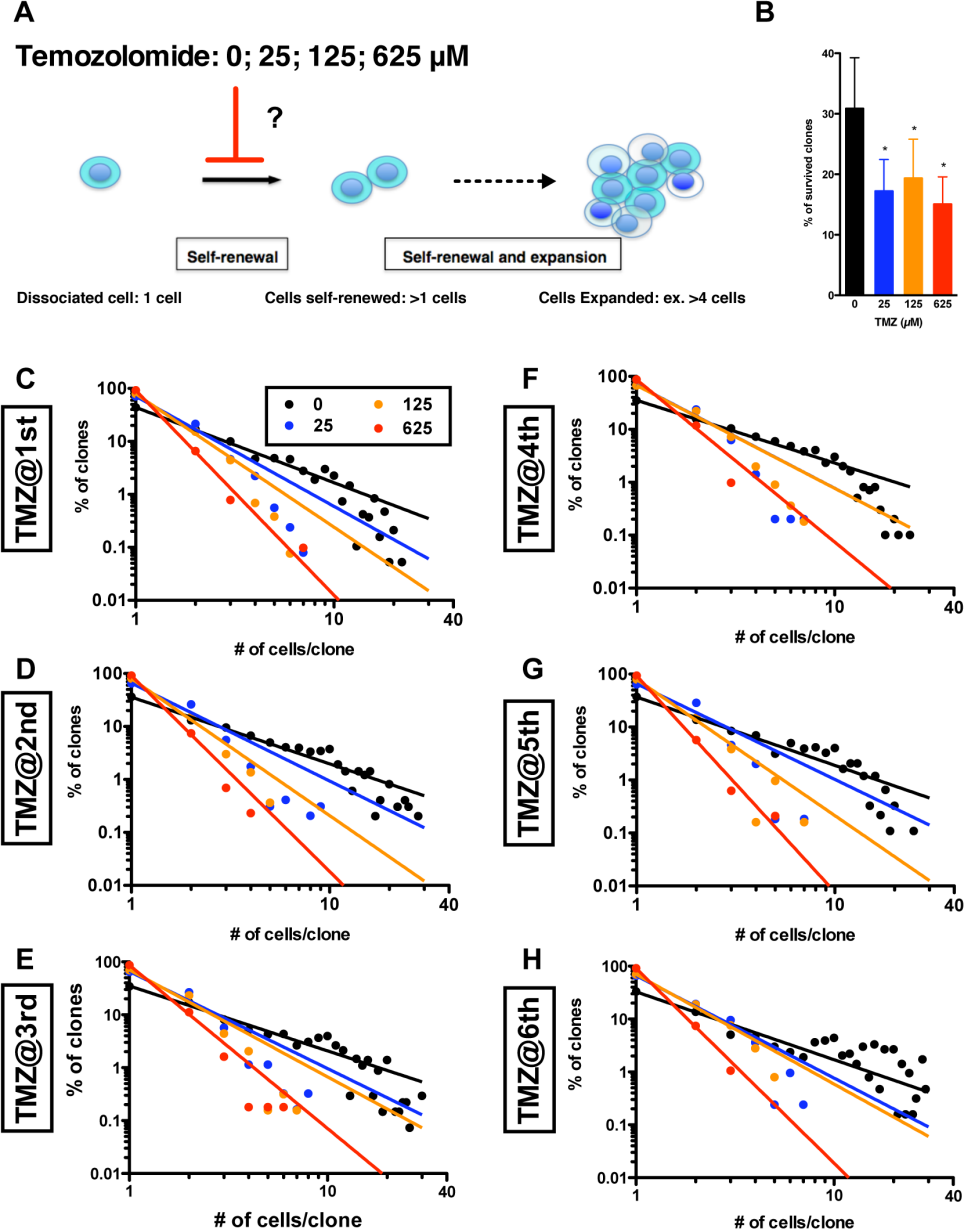
Suppression in growth of U87-derived GS clones in the presence of Temozolomide. (A) U87-derived GS clones were developed in the absence (0 μM, black circle) and the presence of 25 (blue), 125 (orange) and 625 (red) μM of Temozolomide. Temozolomide is a GS-targeting that is thought to suppress survival, potency of self-renewal and clonal expansion of GS clones. (B)-(G) Recapitulation of power-laws in growth of the GS clones in the presence of Temozolomide. The graphs show double logarithmic plot of number of cells/ clone and of the frequency in each generation (1^st^ to 6^th^).

We next examined whether a power-law for growth can be disrupted by a pharmacological agent such as Temozolomide, which is a standard treatment for glioblastoma multiforme, is suggested to inhibit self-renewal of GSCs [32,33]. We first addressed whether Temozolomide can disrupt growth of clones in the development of tumor neurospheres (Fig 5A). We administered 25, 125 and 625 μM of Temozolomide in the methylcellulose-containing assaying culture. We found significant suppression in the growth of U87-derived GS clones at every generation (Fig 5B–5H). However, the distribution of clone sizes showed that GS populations always followed a power-law in the presence of Temozolomide. This suggests that Temozolomide suppresses the self-renewal of GSs independent of clonal diversity and power-law formation.

#### Recovery of growth of U87-derived GS clones while maintaining a power-law after removal of Temozolomide

**Fig 6:**
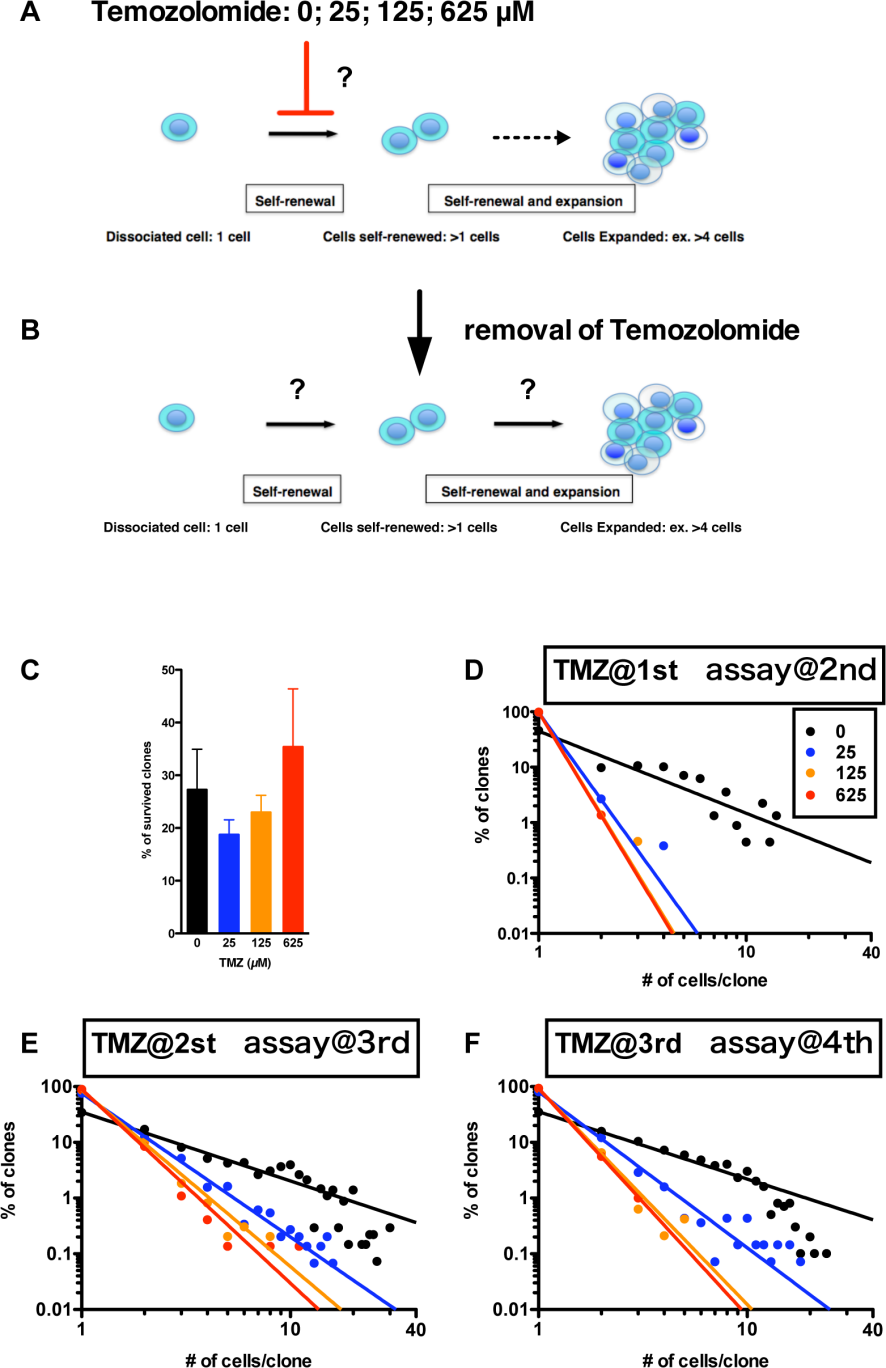
Recovery growth of U87-derived GS clones after administration of Temozolomide. (A) U87-derived GS clones were developed in the absence (0 μM, black circle) and the presence of 25 (blue), 125 (orange) and 625 (red) μM of Temozolomide. Then, the GSs were dissociated and analyzed in the absence of Temozolomide in the each following generation. (B)-(D) Recovery growth of the GSs in the following generations (2^nd^, 3^rd^, 4^th^ for B, C, D) after the Temozolomide administration at the previous generations (1^st^, 2^nd^, 3^rd^ for B, C, D). The graphs show double logarithmic plot of number of cells/ clone and of the frequency in each generation.

We next determined whether U87-derived GS clones could recover growth while maintaining a power-law after removal of Temozolomide. The growth of GS clones was suppressed, especially in the 2nd generation, when Temozolomide had been administered in the 1st generation (Fig 6A and 6B). Growth suppression appeared to have been overcome in the 3^rd^ and 4^th^ generations. While these latter generations became somewhat less diverse, in every experiment, a significant population of GS clones survived while maintaining a power-law (Fig 6C–6F). Thus, the anti-glioma/GSC agent Temozolomide suppressed the self-renewal of clones, but it never disrupted the power-law behavior of a GS population.

## Discussion

### Functional heterogeneity in GSs

Here, we quantified the number of clones and the number of cells in every clone in the tumor neurosphere culture. Our key finding is that GSs generated clonal diversity, and that the frequency distribution of the sizes of clones within a GS population always follows a power-law, with the power constant k ranging between—1.42 and—0.97. The absolute number of the k value indicates how fast the population grows, or, alternatively, how enriched expandable clones are in the population. Thus, clonal diversity and the power-law for growth are characterized by a quantitative measure of heterogeneity, the k value of the GS population.

Previously, GSC heterogeneity has only been described qualitatively, by the expression of marker genes, and these studies suggested that the heterogeneity was generated as a set of hierarchically differentiated GSCs. On the other hand, quantitative heterogeneity in the sizes of clones in benign skin tumors is caused by cell cycle exit and irreversible differentiation [20]. Our data show that GS clones in which clonal cells differentially divide are *functionally* heterogeneous and rather diverse. Diversity in cell number per clone suggests the possibility that cells arrest in cell cycles programmed to different timings. While the formation of different-sized clones is explained by the SC model of tumor maintenance and tissue growth in which the properties of a self-renewal system include a hierarchy of cells having a range of proliferative potential [34], self-renewing capacity must be determined in a heterogeneous CSC population in which subpopulations of CSC clones differentially generate CSCs by symmetric or asymmetric cell division [7].

The question, then, is whether cell divisions within a clone are associated with terminal differentiation [20] or with stem cell maintenance, a self-renewal event. Our data from size-based sub-clone isolation experiments show that larger clones recapitulate the same power-law growth, suggesting that these clones contain infrequently dividing cells. Larger clones also indicate maintenance of cell generation in which symmetric or asymmetric cell division must occur. Moreover, the growth of smaller clones was able to reproduce a power-law, suggesting that the smaller clones did not consist of terminally differentiated cells, but rather of self-maintaining infrequently dividing cells. Thus, the functional heterogeneity shown in this study was not a consequence of irreversible terminal differentiation. Our data for smaller clones also support the idea that GSs maintain their cell division properties for recovery of the characteristic power-law of a GS population. It thus appears that the GS population undergoes self-renewal and maintains power-law behavior.

### Dynamics underlying generation of clones in a population of GSs

Our study shows clonal diversity and a power-law growth pattern in a population of U87-derived GSs, raising two questions: Q1) In the maintenance of diversity and for power-law growth, does such a population depend upon environmental conditions? Q2) How small a population can exhibit clonal diversity and the power-law relationship? We found that a population as small as 200 clones and progressively expanded clone-derived clones both showed similar clonal diversity and a power-law growth pattern. Thus, it is difficult to discern whether inter-clonal or intra-clonal mechanisms underlie clonal diversity. However, it does suggest that within the clone each cell possesses the collective intelligence required for power-law coded GS generation. The power-law growth pattern was recapitulated not only in a population of surviving clones, but also, in a subset of populations of self-renewed clones and expanded clones. Therefore, the data suggest that the underlying mechanism for the power-law growth pattern does not rely on the existence of clones of a specific size [17]. The U87-derived GS population reproduces power-law behavior after exposure to Temozolomide, even when self-renewal is suppressed at the clone level. Thus, the power-law for growth is scale-free, and is based on the collective intelligence and emergent mechanisms of the GSs.

### Self-renewal and plasticity in growth of a heterogeneous population of GSs

If CSCs in a cancer cell population have cell properties that are in stable states and are homogeneous, then CSC maintenance (self-renewal) is the necessary mechanism for recapitulating the CSC population [2,35]. However, in many cancers, CSCs show heterogeneity, raising the question of how heterogeneity in a CSC population is recapitulated over generations [11,18,36].

The present study shows that the GS population is heterogeneous and reproduces the heterogeneity via self-renewal. That repeated passaging of clones recapitulated the heterogeneity of the original SC population and replicated the original power-law-dependence of that heterogeneity, does not support the hypothesis stated in the Introduction.

If a heterogeneous CSC population consists of clones, each with a distinct self-renewal frequency, where CSC clones of different sizes expand at different rates, the CSC clones that expand the most readily would eventually replace the other clones in the CSC population [27,36,37]. This would suggest that the previous heterogeneity is not recapitulated. But this is not consistent with our findings, which indicate that the diversity and the power-law for growth within the GS population are recapitulated during repopulation. Alternatively, if clones of different sizes in a heterogeneous CSC population where all maintain themselves with the same self-maintaining frequency, the heterogeneous population of clones would reappear. However, clones of different sizes would not generate diversity and a power-law for growth in the CSC population. The differential growth of CSCs alone or self-maintenance alone does not explain how a heterogeneous CSC population self-renews. Also, stable states of cell properties of each clone do not explain how heterogeneity is reproduced. This suggests CSC plasticity exists in which clones change their spatial and temporal properties.

Our study showed that clones consisting of either small or large numbers of cells recapitulated clonal diversity over several generations. That is, progressively expanded (large) clones can generate cells that will become small-sized clones, are composed of infrequently-dividing cells. On the other hand, small-sized clone-derived clones are able to become progressively expanded clones, ones that are made of frequently-dividing cells. Even after suppression of self-renewal by Temozolomide, GSs retained power law behavior, supporting the view that GSCs may flexibly and reversibly change their properties in order to re-generate GSC heterogeneity.

### The Scale-Free Power-Law for Growth in the Self-Renewal of a Heterogeneous Population of GSs

We found that heterogeneity was recapitulated, and followed a power-law, in a small population of clones that are spatially restricted. Moreover, this power-law for growth is scale-free in the GS population, indicating that a subset of the GS population is reproduced regardless of the space it initially occupies or of the initial clone sizes.

This reproductive capacity suggests two different models, fractal and scale-free network models [38,39], that could explain our findings. A *fractal model* can explain how a population of GSs maintains the characteristics of the population, independent of the spatial domain where the population resides. A *scale-free network model* can explain how a scale-free power-law is formed and reproduced.

Considering the heterogeneous GS population as a scale-free network model allows us to discuss how CSCs might make a therapeutic target. The scenario in which a heterogeneous CSC population is reproduced over generations, and a power-law is recovered can be interpreted using an analogy to the scale-free network model (containing hubs with many connections and nodes with fewer connections). The heterogeneous CSC population is then considered to be a network where clones/cells/signaling mechanisms could be thought of as nodes. Research on scale-free topology systems shows that the organizing processes acting at each stage of network formation or network evolution are governed by two laws, growth and preferential attachment [40]. In a scale free network, even when nodes are randomly attacked and/or destroyed [41], the network is robust and maintained when hubs are continuously generated. The robustness of a reproducible CSC population may be explained if hub-like nodes exist in the CSC population in accordance with the robustness of a scale-free network. This implies that a cell population in which a scale-free power-law controls the development and the maintenance of the population may be resistant to non-targeting (randomly attacking) pharmaceutical agents or environmental exposures such as anticancer drugs or a hypoxic environment. Conversely, a scale-free network is highly vulnerable when the less frequent hubs are selectively attacked and eliminated. The removal of the hubs causes a drastic change in network topology, and the population of nodes becomes fragmented, which leads to system failure. When the attack is extended to smaller hubs, resulting in their removal, the entire population is destroyed in accordance with the disruption of the power-law. This implies that the scale-free power-law dependent cell population can be functionally disrupted if hubs are selectively or continuously attacked.

We found that larger clones are less frequent and follow a power-law. Are larger clones functionally like hub cells? It could be, because larger clones contain many cells, suggesting much networking and cell-cell interactions happen within a clone. However, we believe that the interventions that target just the larger clones would not work. Our study showed that small clones by themselves can reproduce a heterogeneous, power-law dependent population over multiple passages. Moreover, large clones also contain cells that can generate heterogeneous clones. Thus, the clone sizes probably do not reflect hub or non-hub functions in the population. When just a couple of clones were cultured in a well, we found that some became large but most stayed small (Y. Hayakawa, unpublished data), suggesting that each seeded clone knows how to behave, independent of space, time and number of cells.

This suggests that the mechanisms that control power-law dependent growth and self-renewal are intrinsic to the cell (i.e., they have collective intelligence), suggesting the possibility that disruption of the intrinsic signals that control reproducibility of power-law dependent growth may deplete the entire GS population, or may at least disrupt cellular functions in the self-renewal of the GS population. Thus, mechanisms involved in a fractal and/or a scale-free network system may regulate GS plasticity and the scale-free power-law behavior for growth. Such mechanisms could explain how the heterogeneous GSC population self-renews, as proposed for tissue stem cells [42].

### Dynamics of Cancer Stem Cell Heterogeneity and Self-Renewal

CSC theory dictates that it is essential to identify and understand the traits of homogeneous CSC populations in order to develop anti-cancer therapies that are capable of targeting them. However, our study of baseline GS dynamics involved in heterogeneity raises issues regarding how CSCs function quantitatively. We have discovered key details of a process in which GSs can interconvert between different growth properties (see fractionation experiments) and self-renew while maintaining the heterogeneity of the GS population. Interconversion of SCs and non-SCs has recently been recognized to occur in various normal and malignant tissues [19]. Previous in vivo transplantation studies with specific markers have demonstrated that hundreds to thousands of CSC/GSC populations reproduce the tumor tissue. However, the plasticity, inter-conversion and quantitative diversity of CSC/GSCs during the reproduction of tumor tissue in vivo has remained largely unexplained [35].

Our study can explain not only how a heterogeneous CSC population self-renews, but also how the CSC population generates cancer cells with unrestrained growth. As discussed above, GS plasticity in growth, while following a power-law, could explain how a heterogeneous CSC population self-renews. On the other hand, an increase in size of only expandable clones suggests that an increase in the frequency of production of cells only within expandable clones, without changing the proportion of small sized clones, could explain unrestrained growth of CSCs. This raises the possibility that targeting only progressively expandable CSCs and/or the mechanism controlling the progression, might suppress the expansion of the size of the CSC population. However, even this approach might not suppress heterogeneity of CSCs and the reproducibility of the overall CSC population and cancer growth might persist. Indeed, we found that GS populations can self-renew while reproducing a power-law even after exposure to the glioma/C-targeting agent Temozolomide.

## Conclusions

Our study shows that quantitative analysis (enumerating the number of cells per clone) of tumor SC heterogeneity in the context of self-renewal can provide important information on mechanisms of tumor growth (e.g., the involvement of scale free power law behavior in tumor growth). It suggests that clonogenic systems such as the one we used herein may prove valuable for in vitro screening of potential anti-cancer agents by studying their effects on stem cell self-renewal and heterogeneity [34,43]. Our study also shows that a U87-derived GS population self-renews while reproducing heterogeneity and power-law behavior even after exposure to Temozolomide, even when self-renewal is suppressed at the clone level. This suggests that self-renewal of a population is concomitantly regulated and supported by a mechanism which controls a power-law. If targeting a heterogeneous CSC population is to be developed as a therapeutic strategy, as our findings suggest it will be crucial to determine the molecular mechanisms underlying scale-free power-laws in which a CSC population reversibly self-renews and repopulates itself. We believe that having this knowledge will lead to the development of strategies that target the plasticity of CSC populations and would efficiently prevent recurrence of cancers and possibly help cure them altogether [44,45].

## Supporting Information

S1 Fig Self-renewal and expansion of the GS clones in the tumor neurosphere culture. The graphs show: i). Survival of the clones of U87 (black circle), U251 (red), A172 (blue) and T98 (orange) cell-line derived GS population based on the number of clones (A)-(E); ii). The percentage of various sized clones in the total surviving clones (F)-(I), in the self-renewed clones (J)-(K) and in the expanded clones (L); iii).The average growth based on average number of cells/clone (M)-(P). The data were derived from populations of the total surviving (1–40 cells for A, F, M), single-cell (1 cell for B, F), self-renewed (2–40 cells for C, G, N) and expanded (5–40 cells for D, H, J,O; 10–40 cells for E, I, K, L, P) clones. Data for each graph were derived from 3 to 5 independent experiments.
(EPS)

S2 Fig Self-renewal and expansion of the GS population in the tumor neurosphere culture. The graphs show: i). The size (the number of cells) in populations of U87 (black circle), U251 (red), A172 (blue) and T98 (orange) cell-line derived GS population based on the number of clones (A)-(E); ii). The percentages of cells derived from various sized clones in total surviving populations (F)-(I), in the population of self-renewed clones (J)-(K) and in the population of expanded clones (L). As shown in (F)-(I), the size of the population gradually expanded especially in U87 and U251 derived population. The graphs show increase in the percentage of multicellular clones. Self-renewed and expanded clones occupied larger percentages of cells than of clones in surviving population. The data were derived from populations of the total surviving (1–40 cells for A), single-cell (1 cell for B, F), self-renewed (2–40 cells for C, G) and expanded (5–40 cells for D, H, J; 10–40 cells for E, I, K, L) clones. Data for each graph were derived from 3 to 5 independent experiments
(EPS)

S3 Fig Normalized GS populations exhibit diversity and follow a power-law for growth during repopulation. (A) The double logarithmic plot for the frequency distribution of clones with different size (number of cells per clone). The size was normalized, whereby the number of cells per clone was divided by the average of number of cells per clone. (B) The table shows the CV values for the populations shown in the above.
(EPS)

S4 Fig Normalized GS populations exhibit diversity and follow a power-law in growth in both large-sized and small-sized clone-derived populations. (A) The double logarithmic plot for frequency distribution of clones of different sizes (number of cells per clone). The size was normalized, whereby the number of cells per clone was divided by the average of number of cells per clone. (B) The table shows the population CV values for each separate population.
(EPS)
